# Assessing cancer knowledge among pharmacy students in Jordan: Bridging the gap between theory and practice

**DOI:** 10.1371/journal.pone.0327187

**Published:** 2025-07-28

**Authors:** Ali Hmedat, Haneen Amawi, Ghaith Al-Taani

**Affiliations:** 1 Department of Pharmaceutics and Pharmaceutical Technology, Faculty of Pharmacy, Yarmouk University, Irbid, Jordan; 2 Department of Clinical Pharmacy and Pharmacy Practice, Faculty of Pharmacy, Yarmouk University, Irbid, Jordan; Northern Border University, SAUDI ARABIA

## Abstract

**Background:**

Early detection of cancer is essential in improving patients’ clinical outcomes. As future first-line health providers, pharmacy students must possess strong knowledge and education regarding cancer risk factors, early signs and symptoms, and available screening methods. The current study aimed to assess knowledge among pharmacy students at various Jordanian universities concerning key cancer related topics. This study particularly assessed the students’ knowledge and awareness of cancer signs and symptoms, associated risk factors, and possible screening methods.

**Methods:**

This cross-sectional study was conducted from June to September 2024, using an online survey among pharmacy students from various Jordanian schools to assess their knowledge about cancer and its warning signs, risk factors and screening recommendations. The questionnaire was distributed electronically through student networks and in collaboration with pharmacy schools across Jordan. Participation was voluntary, and students were included based on their consent to participate. Data analysis was performed using the Statistical Package for Social Sciences (SPSS) version 26. Bivariate correlations were assessed using Spearman’s rank correlation. Additionally, simple, multiple, and backward linear regression analyses were conducted to identify significant predictors of cancer knowledge.

**Results:**

More than 60% of pharmacy students considered cancer education adequate in their programs. However, around half reported insufficient knowledge about the management of cancer patients and safe handling of anticancer medicines. Few of respondents correctly identified warning signs of cancer, with the mean summated score of 6.1 out of the maximum possible 12. The mean summated score equaled 7.8 out of 14 maximum possible scores about cancer risk factors. Linear regression analyses showed that students without a personal history of cancer and those who believe it is important to have oral chemotherapy knowledge have increased knowledge about cancer related topics. Also, those interested in continuing education programs on oral chemotherapy have higher knowledge about cancer warning signs.

**Conclusion:**

The results revealed gaps in practical cancer education and the knowledge in cancer risk factors and warning signs among pharmacy students. Thus, enhanced oncology education is essential to better prepare pharmacy students to contribute effectively to multidisciplinary cancer care teams.

## Introduction

The continuously increasing rate of cancer incidence, prevalence and related deaths is alarming. According to the latest World Health Organization statistics in 2022, there were 20 million new cancer cases worldwide [1]. Despite the improved therapeutic tools in screening, diagnosis and management of cancer, there were 9.7 million cancer-related deaths with approximately 1 in 9 men and 1 in 12 women dying from the disease in the same year [1].

While these global figures are alarming, the rising cancer burden is also evident at the national level, with Jordan experiencing a similar upward trend in incidence. However, comprehensive data on cancer statistics in Jordan remain limited, highlighting the need for more frequent and detailed national reporting. The last governmental report from the Jordan Cancer Registry (JCR) was in 2022 and reported significant increase in the incidence of cancer with 10,775 new cases, with Age Standardized Incidence Rate (ASR) adjusted to the World Standard Population being 157.4 per 100,000 population [2]. The top 5 reported cancers from the same report were breast, colorectal, lung, lymphoma, and bladder [2]. Similarly, the latest 2022 report from the Global Cancer Observatory (GLOBOCAN), reported approximately 12,328 new cancer cases in Jordan, with breast, colorectal, and lung cancers being the most commonly reported in both males and females [3].

Most cancers are curable and preventable if diagnosed at early stage. Early diagnosis is associated with less invasive treatments and improved outcomes [4]. Unfortunately in Jordan, current early screening programs are limited except for breast cancer, and most cancers are diagnosed at locally advanced or metastatic stages [5]. Thus, early and alarming signs and symptoms, such as rectal bleeding and breast lumps should be well recognized by public and general health care providers to prevent further delay in diagnosis.

Risk factors for different cancer diseases are variable. However, a sedentary lifestyle, high consumption of processed food, obesity, smoking, air pollutants, and alcohol consumption are among the most reported factors globally [6, 7]. Smoking and obesity are the top risk factors in Jordan with the country ranking among the highest smoking rates in the region, especially among young females and schoolchildren [5]. Thus, there is an urgent need for effective awareness campaigns to promote smoke cessation, lifestyle modifications, including increased physical activities, healthy dietary habits, and weight loss.

Pharmacists are accessible frontline health care providers and should be well-educated and prepared to provide their patients with multidisciplinary interventions and beneficial facts about cancer. Therefore, evaluating cancer knowledge and awareness among pharmacy university students is essential. Previous studies have reported insufficient knowledge about cancer-related topics among pharmacy students [8–11], but most of these investigations were conducted in other countries or focused on limited aspects of cancer education. In Jordan, existing studies have highlighted the role of practicing pharmacists, focusing on their understanding of cancer warning signs and screening methods [12–15]. However, there is a noticeable gap in research exploring this subject among pharmacy students.

The current study aimed to assess the pharmacy students’ knowledge in several universities in Jordan regarding cancer-related topics. It is specifically assessed the students’ understanding of cancer’s signs and symptoms, risk factors, and screening methods.

## Methodology

### Study design

A cross-sectional online survey of pharmacy students in different pharmacy schools in Jordan assessed the quantitative knowledge about cancer and its warning signs, causes, risk factors and screening recommendations over the period June to September 2024. The distribution was carried out online using student networks and connections with different pharmacy schools in Jordan employing convenience sampling. Participating pharmacy students were selected based on their consent to take part in the survey. At the beginning of the study, they confirmed that they were enrolled in a pharmacy program.

### Ethical considerations

The Institutional Review Board (IRB) research ethics committee at Yarmouk University approved this study under the approval number **(IRB/2024/251).** A written informed consent form was provided at the beginning of the electronic survey questionnaire, and only students who gave informed consent were permitted to proceed with the questionnaire and participate in the study. No minors were included in this study.

### Data collection

We developed a survey to assess pharmacy students’ knowledge of cancer, cancer recognition, cancer risk factors and cancer screening recommendations. The main parts of the survey came from tools that were published in research journals in the area [16, 17] with some small changes made by the research team to make them more useful for pharmacy students. These published surveys were carefully developed, underwent rigorous faculty review for content validity and were deemed statistically reliable [16, 17]. The developed survey was then subject to review by faculty members from several pharmacy schools in Jordan to assess the content validity. Content validity index (CVI) was calculated after a validation exercise of 4 reviewers who are faculty members with PhD in pharmacy with interest in this research topic. Results revealed overall scale CVI of 87%. For the subscales, the CVI scores were as follows, 97.9% for the demographic section, 93.2% for the knowledge, 75% for the risk factors, 77.1% for the warning signs and 100% for the screening recommendations. We piloted the questionnaire among 10 pharmacy students and solicited their comments. Any comments from the faculty members or the pilot distribution were considered, and minor changes were made to the survey, mainly on the overall format and length of the survey.

The survey consisted of six parts. Part 1 was concerned with demographic and background information (7 items) that included the gender, oncology and chemotherapy education received within the degree program, personal and family history of cancer, and their opinion about the importance of pharmacists knowing about oral chemotherapy drugs and their interest in completing a continuous education program on oral chemotherapy. Part 2 (2 items) was designed to capture the didactic aspect about oncology medicine use, including teaching received and training needs. Part 3 assessed the knowledge of cancer using 10 factual knowledge statements in which the pharmacy student selected the options: true statement, false statement, or I don’t know (10 items). For all scales, a summated score was calculated for each participant by summing the number of correct answers. Part 4 assessed participants’ knowledge of cancer signs and symptoms through 12 items, resulting in a similar summated score. Part 5 measured the students’ knowledge about the risk factors of cancer reporting individual and summated scores (14 items). Part 6 asked about the student’s knowledge of cancer screening recommendations, looking at the individual statement and summated score (7 items).

Internal consistency was assessed using Cronbach’s alpha for each section of the questionnaire, yielding the following values: 0.754 for general cancer knowledge, 0.849 for knowledge of cancer warning signs, 0.825 for knowledge of cancer risk factors, and 0.869 for knowledge of cancer screening recommendations. The questionnaire was uploaded on Microsoft Forms and distributed online. Participants for this study were recruited between 10.06.2024 and 11.9.2024. During this period, the survey was distributed using online platforms and via faculty members within different faculties of pharmacy in Jordan. The distribution was operationalized through an invitation to complete the survey, which includes information about the study and its purpose, the target population, the fact that participation is voluntary and anonymous, the fact that participants can withdraw from the study at any time, and the link to the Microsoft forms for completion.

We used the first page of the survey for initial screening, excluding participants who indicated they were not pharmacy students. Also, on the first page, a consent form was embedded; those who did not agree to participate were directed out of the form. The setting for the responses form was set as “required” and “only one response”, to maximize the quality of the responses and facilitate the distribution. Recruitment was planned until the achievement of the predefined sample size that equaled 377 responses. The required sample size was calculated using the Raosoft online sample size calculator [18], assuming a total population of 10,000 pharmacy students in Jordan, a 95% confidence level, a 5% margin of error, and a response distribution of 50%.

### Data analysis

Statistical methods have been used to achieve the study objectives. The analysis was run using the Statistical Package for Social Sciences (SPSS) version 26. Numbers, frequencies, means and standard deviations were descriptive statistics used to summarize the main trends within the data. Bivariate correlation analyses among the summated scales within the study were carried out using the Spearman correlation technique. Multiple analyses were carried out to assess the factors associated with different summated scales. Initially, a simple linear regression analysis was made to assess the candidate variable to be included in the final linear regression model, candidate variables were those with P < 0.250. Then, those candidate variables were included in backward linear regression analysis, retaining only independent predictors associated with each summated scale. The backward linear regression model produces the smallest number of predictors that best explain the outcome of interest. It is often preferred over other methods due to its favorable performance metrics and is widely used in healthcare research [19]. Statistical significance is set at p ≤ 0.05.

## Results

The present study was conducted over the period June to September 2024 and achieved 422 responses, which was above the estimated minimum sample size calculated. Table 1 summarizes the trends with demographic and background variables for the study sample. Participants were recruited from five pharmacy schools in Jordan: Yarmouk University, Jordan University of Science and Technology, Petra University, Isra University and Al Zarqa University. There have been more female respondents (N = 346, 82%) compared to males. Most (N = 263, 62.6%) of the respondents indicated adequate education concerning oncology and chemotherapy in their degree program. One-third or less of the respondents reported having a family or personal history of cancer. The respondents expressed positive beliefs regarding the importance of knowledge about oral chemotherapy drugs and their preference for continuing a continuous education program on oral chemotherapy.

**Table 1:** Demographic and background variables.

Variable		N (%)
Gender	Male	76 (18.0)
	Female	346 (82.0)
Pharmacy degree	Pharm D	141 (33.4)
	BSc	281 (66.6)
University	Private	19 (4.5)
	Public	403 (95.5)
Year of study	3 or less	113 (26.8)
	4	14 (3.3)
	5 or later	295 (69.9)
Adequate oncology education during undergraduate degree	Yes	284 (67.5)
	No	137 (32.5)
Received adequate chemotherapy education during undergraduate degree:	Yes	263 (62.6)
	No	157 (37.4)
Personal history of cancer	Yes	57 (13.6)
	No	362 (86.2)
Family history of cancer	Yes	128 (30.5)
	No	253 (60.2)
	I don’t know	39 (9.3)
Importance of having good knowledge of oral chemotherapy drugs by the pharmacist	1. Not Important.	18 (4.3)
	2	24 (5.7)
	3	75 (17.8)
	4	76 (18.0)
	5. Very Important.	229 (54.3)
Interest in in completing a continuing education program on oral chemotherapy	1. Not Interesting.	27 (6.4)
	2	39 (9.2)
	3	114 (27.0)
	4	89 (21.1)
	5. Very Interesting.	153 (36.3)

Concerning the teaching received about oncology, more than two-thirds of respondents reported adequate teaching in pathophysiology (N = 322, 76.3%) and pharmacology (N = 295, 69.9%). In contrast, about half of respondents reported receiving adequate teaching about the management of patients with cancer (N = 201, 47.6%) and safe practices to handle anticancer medicines (N = 217, 51.4%). A significant proportion of respondents reported that they needed more information about pharmacology (N = 287, 68.0%), how to use (N = 256, 60.7%) and prescription anticancer medicines (N = 245, 58.1%), and types of cancer (N = 250, 59.2%). Table 2 illustrates these trends.

**Table 2:** Teaching received and training needs about oncology medication use.

Have you had any teaching about oncology medication use during your undergraduate degree?			
Teaching received	Yes N (%)	No N (%)	Unsure N (%)
Pathophysiology of cancer	322 (76.3)	53 (12.6)	47 (11.1)
Pharmacology of anticancer medicines	295 (69.9)	81 (19.2)	46 (10.9)
Management of patient with cancer	201 (47.6)	146 (34.6)	75 (17.8)
Safe practices to handle anticancer medicines	217 (51.4)	135 (32.0)	70 (16.6)
**On which topics would you like to receive more information?**			
**Training**	**N (%)**		
Pharmacology of anticancer medicines	287 (68.0)		
How anticancer medications are used	256 (60.7)		
Types of cancer	250 (59.2)		
Prescription of anticancer medications	245 (58.1)		
None	34 (8.1)		

Responding pharmacy students scored high on knowledge about cancer, with a mean summated score of 7.5 out of 10, the maximum possible score. The statement that cancer occurs when abnormal cells divide uncontrollably received the highest score (N = 391, 92.9%). The lowest score achieved was for that cancer is just one disease (false), which was correctly answered by 68.9% of the participants (N = 290). Table 3 has the full details regarding the knowledge assessment about cancer. Moving to the responding pharmacy students’ knowledge about warning signs of cancer, a considerably lower proportion of respondents provided correct responses on these items. Indeed, the mean summated score was 6.1 out of a maximum possible score of 12. Less than 50% of the respondents correctly answered many of the statements (n = 7). The highest score was achieved for unusual lump (N = 337, 80.0%), followed by the change in the size of a mole or wart (N = 270, 64.1%). Refer to Table 4 for a more detailed description of these knowledge items.

**Table 3:** Assessment of the pharmacy students’ knowledge about cancer.

Knowledge statement	N (%) correct
Cancer is when abnormal cells divide in an uncontrolled way (True)	391 (92.7)
Cancer is just one disease (False)	290 (68.9)
Cancer cells can also spread to other parts of the body (True)	389 (92.8)
There are few types of cancer (False)	294 (69.8)
Cancers are usually named for the organs or tissues where the cancers form (True)	373 (88.4)
Anybody is susceptible to cancer (True)	344 (81.7)
Cancer is not life-threatening disease (False)	325 (77.0)
Cancer is an infectious disease (False)	305 (72.3)
The most common treatments for cancer are surgery, chemotherapy and radiation (True)	388 (91.9)
Early detection of cancer is difficult because there are no specific symptoms (False)	304 (72.0)
**Total knowledge subscale descriptive statistics**	
Maximum possible score	10
Mean	7.5
Standard deviation	1.9

**Table 4:** Assessment of student pharmacists’ knowledge about warning signs of cancer (all true).

Warning sign	N (%) correct
A new or unusual growth, lump or swelling anywhere on the body	337 (80.0)
Sore throat that does not heal	171 (40.6)
Changes in the shape or size of a mole or wart	270 (64.1)
Blood in urine or stool	240 (57.0)
Unusual bleeding or discharge from the nipple or vagina	233 (55.3)
Change in bowel or bladder habits	203 (48.2)
Difficulty in swallowing	180 (42.8)
Indigestion	148 (35.2)
Unexplained loss in weight	246 (58.4)
Unexplained fever, tiredness or pains	203 (48.2)
Persistent or recurrent infection	170 (40.3)
Joint inflammation	169 (40.1)
**Total warning signs knowledge subscale descriptive statistics**	
Maximum possible score	12
Mean	6.1
Standard deviation	3.6

Knowledge of cancer risk factors was the subject of Table 5. The mean summated score equaled 7.8 out of 14 maximum possible score. The most correctly answered risk factor was smoking (N = 373, 88.4%). Issues were noted regarding overweight and obesity, insufficient physical activity, unbalanced diet, infections, hormones, and lack of fruit and vegetable intake. Table 6 assessed pharmacy students’ knowledge of cancer screening recommendations and revealed a high proportion of correct responses, ranging from 64.3% to 77.4% across individual items, with a summated score of 4.9 out of 7, the maximum possible score.

**Table 5:** Assessment of student pharmacists’ knowledge about risk factors of cancer.

Cancer risk factor	N (%) correct
Smoking (True)	373 (88.4)
Radiation exposure (True)	334 (79.1)
Overweight and obesity (True)	148 (35.1)
Insufficient physical activity (True)	122 (28.9)
Excessive alcohol intake (True)	280 (66.4)
Unbalanced diet (True)	136 (32.2)
Vaccine (False)	318 (75.4)
Drink a lot of coffee (False)	351 (83.2)
Deodorant or antiperspirant use (False)	254 (60.2)
Migraines (False)	338 (80.1)
Infections (True)	130 (30.8)
Hormones (True)	189 (44.8)
Chronic inflammation (True)	207 (49.1)
Lack of fruits and vegetable intake (True)	109 (25.8)
**Total cancer risk factors knowledge subscale descriptive statistics**	
Maximum possible score	14
Mean	7.8
Standard deviation	2.2

**Table 6:** Assessment of the pharmacist students’ knowledge about cancer screening recommendations (all true).

Cancer screening recommendation	N (%) correct
It is recommended that women aged 20–30 years undergo clinical breast examination periodically (every 3 years).	322 (77.4)
Women aged 40 years and above should undertake mammography annually.	308 (74.4)
Women aged 30–65 years should undertake HPV test and Pap test every 5 years.	265 (64.3)
Men and Women aged 50 years and above should undertake fecal occult blood test annually.	262 (63.4)
Men aged 50 years at high risk should undertake digital rectal examination annually.	274 (66.7)
At the time of menopause, women at average risk should be informed about risks and symptoms of endometrial cancer.	290 (70.4)
Women are strongly encouraged to report any unexpected bleeding or spotting to their physicians.	300 (72.5)
**Total cancer screening recommendations knowledge subscale descriptive statistics**	
Maximum possible score	7
Mean	4.9
Standard deviation	2.3

The present study also assessed the relationship between different scales using bivariate correlations, and it has been found that there have been weak to moderate correlations between different scales (Spearman correlation coefficient > 0.196) and all of them had statistically significant associations (P < 0.001) (Table 7).

**Table 7:** Bivariate correlation of the total of the subscales.

Variable	Spearman correlation coefficient			
	Total knowledge subscale	Total warning signs knowledge subscale	Total cancer risk factors knowledge	Total cancer screening recommendations knowledge subscale
**Total knowledge subscale**	**–**	0.264^*^	0.295^*^	0.196^*^
**Total warning signs knowledge subscale**	0.264^*^	–	0.532^*^	0.221^*^
**Total cancer risk factors knowledge**	0.295^*^	0.532^*^	–	0.239^*^
**Total cancer screening recommendations knowledge subscale**	0.196^*^	0.221^*^	0.239^*^	–

*p ≤ 0.001.

Several linear regression analyses were carried out for different knowledge subscales, which are summarized in Table 8. For the knowledge about cancer, those without personal history of cancer and those who belief it is important to have oral chemotherapy knowledge were independent predictors of increased knowledge about cancer. In relation to the warning signs knowledge, those who are interested in completing a continuing education program on oral chemotherapy had higher knowledge about cancer warning signs. Regarding cancer risk factors knowledge, male student pharmacists and those who believe it is important to have oral chemotherapy knowledge were independent predictors of increased cancer risk factors knowledge. Finally, people who learned enough about chemotherapy while they were undergraduates and wanted to continue their education by taking a course on oral chemotherapy were independent predictors of knowing more about cancer screening recommendations.

**Table 8:** linear regression models for different knowledge subscales.

Knowledge about cancer			
Predictor	B* (standard error)	P value	CI for B
Constant	4.566 (0.548)	<0.001	3.489 - 5.643
No personal history of cancer	1.024 (0.269)	<0.001	0.495 - 1.554
Importance of oral chemotherapy knowledge	0.248 (0.080)	0.002	0.091 - 0.405
**Warning signs knowledge**			
**Predictor**	B* (standard error)	**P value**	CI for B*
Constant	3.607 (0.548)	<0.001	2.530 - 4.683
Interested in completing a continuing education program on oral chemotherapy	0.668 (0.140)	<0.001	0.392 - 0.943
**Cancer risk factors knowledge**			
**Predictor**	B* (standard error)	**P value**	CI for B*
Constant	6.860 (0.607)	<0.001	5.667 - 8.054
Female gender	−0.647 (0.275)	0.019	−1.189 - −0.106
Importance of oral chemotherapy knowledge	0.512 (0.092)	<0.001	0.331 - 0.693
**Cancer screening recommendations knowledge**			
**Predictor**	B* (standard error)	**P value**	CI for B*
Constant	4.712 (0.474)	<0.001	3.780 - 5.644
Did not receive adequate chemotherapy education during undergraduate degree	−0.515 (0.236)	0.030	−0.979 - −0.051
Interested in completing a continuing education program on oral chemotherapy	0.235 (0.094)	0.013	0.050 - 0.420

*standardized coefficient.

## Discussion

The prevalence of cancer in Jordan has been consistently rising, mirroring global trends and local demographic changes [20]. The International Agency for Research on Cancer (IARC) forecasts that by 2040, the annual incidence of new cancer cases in Jordan may increase to 21,509 as a result of population expansion and aging.

Hospital oncology pharmacists and community pharmacists play a crucial role in addressing cancer, which has emerged as a significant public health issue [21,22]. Pharmacists are increasingly participating in cancer care by providing education on chemotherapy handling, managing side effects, and offering patient counseling. The prevalence of cancer indicates a need for an increase in the number of pharmacists specializing in oncology. Assessing cancer knowledge and awareness among university pharmacy students serves as an important mechanism for enhancing cancer prevention, early detection, and survival rates. In this context, understanding cancer risk factors and recognizing symptoms are interconnected processes.

Our study focused on the importance of cancer education among pharmacy students in Jordan by evaluating their knowledge of cancer warning signs, symptoms, risk factors, and screening methods. Limited research exists on this topic among pharmacy students in Jordan. Our findings revealed that students demonstrated poor knowledge regarding the warning signs and risk factors of cancer. However, their understanding of cancer screening recommendations was deemed acceptable. Despite this gap in awareness, a majority of participants perceived their oncology education as adequate, highlighting a potential disconnect between what they learned in university and what they really know. This emphasizes the need for enhanced educational initiatives to bridge knowledge gaps in this critical field.

Our study highlights both strengths and gaps in the oncology education of pharmacy students in Jordan. The mean knowledge score of 7.5/10 suggests that pharmacy students generally possess adequate theoretical knowledge about cancer. The high percentage of participants reporting adequate teaching in pathophysiology and pharmacology may reflect their solid baseline understanding of the biological and pharmacological aspects of cancer. However**,** approximately half of the respondents indicated receiving sufficient training in patient management and the safe handling of anticancer medications, highlighting significant gaps in the practical aspects of oncology education. Similar findings have been reported in studies from Saudi Arabia and Turkey, where pharmacy students showed adequate theoretical knowledge but lacked training in practical aspects of oncology care, such as handling cytotoxic drugs or patient counseling [23,24]. This trend appears to extend beyond pharmacy education; for example, a study conducted across six Jordanian universities found that medical students demonstrated only moderate knowledge about cervical cancer, further emphasising the need for improved cancer education across healthcare disciplines [25].

The findings underscores the need to enhance practical and applied education, particularly in managing cancer patients and safely handling anticancer medications, by expanding pharmacy curricula to include more comprehensive oncology-related components. A quick review of pharmacy curricula from selected Jordanian universities involved in our study revealed that oncology is not covered in depth, with most cancer-related content embedded within general pharmacology or pathology courses. This supports our findings and may partially explain the observed gaps in students’ knowledge, particularly in practical and applied oncology topics. Comparable curriculum limitations have also been noted in pharmacy programs across other countries, reinforcing the global need for dedicated oncology courses [26,27]. Addressing these curriculum gaps is especially important given the critical roles of pharmacists in oncology care, where they are expected to contribute to patient counseling, therapy monitoring, and ensuring medication safety [28]. Limited education in these practical areas may hinder pharmacy students’ ability to address real-world oncology challenges, such as advising patients on treatment side effects or managing hazardous drug handling procedures [29].

Our results suggest that students feel underprepared for tasks involving oral chemotherapy, such as educating patients about proper usage and managing side effects. This finding is consistent with studies in other countries, where pharmacy students and even practicing pharmacists have reported limited confidence in handling oral chemotherapy and a need for more hands-on education [30,31]. In our study, the preference among pharmacy students for continuous education programs on oral chemotherapy aligns with these identified gaps in practical oncology training. The expressed interest in continuing education programs on oral chemotherapy reflects students’ awareness of the importance of addressing these deficiencies to be better prepared for real-world oncology practice. It also may show that they are taking charge of their professional growth, which is especially important in this field where pharmacists are becoming important members of cancer care teams. Integrating workshops or seminars on oral chemotherapy into pharmacy curricula may help bridge the gap between theory and practice [32].

The findings regarding pharmacy students’ knowledge of cancer warning signs reveal significant deficiencies, aligning with earlier observations about gaps in practical oncology education. The mean summated score was 6.1 out of 12, with less than 50% of respondents answering many items correctly. These low scores may be attributed to limited clinical exposure during training, insufficient emphasis on oncology content within the curriculum, and a lack of practical, case-based learning opportunities that reinforces early symptom recognition. Similar deficiencies have been reported in previous studies, where pharmacy and other healthcare students demonstrated poor awareness of early cancer symptoms [11,33]. For example, pharmacy and nursing undergraduates attending King Saud University in Riyadh, Saudi Arabia, showed varying levels of awareness of lung cancer symptoms, including cough, weight loss, chest infections, and coughing up blood, among others [9]. Likewise, in Pakistan, the knowledge score of university students regarding the warning signs of colorectal cancer was 59.9%, indicating substantial gaps in recognizing critical early indicators [34].

A lack of knowledge about basic warning signs such as persistent or recurrent infection, unexplained fever or pain and abnormal bleeding could restrict the ability of future pharmacists to identify potential cancer cases and provide timely guidance to patients [35]. The fact that few items, like unusual lump, changes in a mole or wart and unexplained loss in weight were correctly identified by most respondents suggests that students are more familiar with visually apparent symptoms but may lack awareness of systemic or less overt warning signs. This trend is consistent with international findings showing that students tend to recall common visible symptoms more readily than systemic ones [33,36]. Pharmacists are often the first point of contact for patients and may play a critical role in cancer prevention and early detection [37]. Limited knowledge of warning signs could lead to missed opportunities for early referrals and interventions. The results suggest that pharmacy education in Jordan may not currently equip students to serve as effective advocates for cancer awareness within their communities. Enhancing students’ awareness of warning signs is vital for fostering proactive cancer screening efforts and improving early detection rates [38]. Incorporating case-based learning and oncology-focused clinical exposure during internships could help bridge the gap between theoretical knowledge and practical understanding [39–41].

The results from Table 5 reveal a moderate level of knowledge among pharmacy students about cancer risk factors, with a mean score of 7.8 out of a possible 14. The high recognition of smoking as a leading cause of cancer reflects effective awareness campaigns and educational efforts emphasizing smoking as a well-documented risk factor [42]. This aligns with global trends where smoking is commonly recognized as a primary preventable cause of cancer, particularly lung and head and neck cancers [43]. Similar findings have been reported in previous studies, where students demonstrated relatively high awareness of smoking-related risks but lower recognition of other risk factors [8,10,23,34]. For instance, health-related students at Umm Al-Qura University in Saudi Arabia showed poor knowledge of lung cancer risk factors [8]. Likewise, 4th and 5th year undergraduate pharmacy students in Nigeria exhibited only fair knowledge and awareness of breast cancer risk factors and screening methods [10]. In Pakistan, university students demonstrated limited knowledge of colorectal cancer risk factors, with an average score of 40% [34]. Similarly, female pharmacy students in Turkey exhibited a poor understanding of breast cancer risk factors, although their awareness of associated symptoms was relatively acceptable [23].

However, knowledge gaps remain regarding lifestyle-related risk factors like obesity, physical inactivity, and unbalanced diets. This suggests insufficient emphasis on holistic cancer prevention in the curriculum, a problem that is also noted in other studies assessing health sciences students’ knowledge of cancer risk profiles [44,45]. Additionally, inadequate awareness about the role of infections and hormonal factors in cancer development has also been observed in similar populations [11]. These findings point to the need for more comprehensive education on biological and environmental contributors to cancer. Without a thorough understanding of diverse cancer risk factors, pharmacists’ ability to counsel patients effectively on cancer prevention may be limited. Strengthening curricula with an integrated focus on cancer risks would enhance the pharmacist’s role in public health and cancer prevention efforts [46].

Our results show that pharmacy students have a relatively good understanding of cancer screening recommendations, with correct response rates ranging from 64.3% to 77.4% across different items (Table 6). This suggests familiarity with general screening guidelines, which is encouraging given the importance of early detection in cancer management [47]. Similar findings have been observed in studies conducted among pharmacy students in Saudi Arabia and Nigeria, where moderate awareness of screening guidelines was reported [4,10].

This level of knowledge is essential, as pharmacists are frontline healthcare providers who can advocate for cancer screening and educate patients about evidence-based recommendations [36]. However, the fact that no item exceeded a 77.4% correct response rate suggests that students may not fully understand screening protocols, especially for cancers that have specific age, gender, or risk factor-based recommendations. This echoes previous studies indicating that students often possess general awareness but struggle with detailed or less commonly emphasized screening criteria [48].

These findings complement our earlier observations about the need for strengthened oncology education, particularly in applied and patient-focused aspects. Integrating cancer screening guidelines into the curriculum through interactive methods like case studies or clinical simulations has been shown to improve students’ understanding and confidence [49]. By enhancing their understanding of cancer screening, pharmacy students can effectively dispel common patient misconceptions and promote adherence to recommended screening practices [17, 50].

Data analysis revealed weak to moderate relationships between different knowledge scales, as indicated by a Spearman correlation coefficient greater than 0.196 (Table 7), suggesting that knowledge and education variables are interconnected [51]. These findings imply that enhancing education or knowledge in one domain, like cancer warning signs might positively influence related areas like cancer screening recommendations and cancer risk factors (Table 7). This interconnectedness suggests that integrated educational strategies could yield broad benefits.

This study also revealed notable findings through linear regression analyses conducted across various knowledge domains (Table 8). The results showed that basic education and a desire to keep learning are significant predictors of cancer-related knowledge among pharmacy students. For instance, participants who recognized the importance of understanding oral chemotherapy demonstrated significantly higher knowledge scores, particularly about cancer and its risk factors. This likely reflects a proactive mindset where individuals recognize the significance of staying informed about advancements in cancer treatments, which enhances their overall knowledge. Interestingly, students without a personal history of cancer were also more likely to have greater knowledge about cancer. A similar finding was reported by Almutairi et al. (2019), where students with no direct experience of cancer exhibited greater general knowledge, possibly due to stronger academic motivation or broader curiosity about disease prevention, rather than focusing on specific personal or familial experiences [48]. Moreover, students who expressing interest in further education on oral chemotherapy were more likely to have higher knowledge about cancer warning signs and cancer screening recommendations. In summary, our results show that having basic knowledge combined with a willingness to pursue ongoing learning, significantly contributes to higher levels of cancer-related knowledge among pharmacy students.

Although our study produced better results than previous research [8,10,23,34,52], it indicates that we have not yet reached the ideal knowledge level about cancer. Therefore, pharmacy education must be revised to provide a more comprehensive and in-depth understanding of cancer, and pharmacy students should be required to take an oncology pharmacy course to further broaden their knowledge.

### Strengths and limitations of the study

To the best of our knowledge, this is the first study in Jordan to exclusively investigate the level of cancer knowledge among pharmacy students. This unique focus provides valuable insights into an understudied group, highlighting specific educational gaps and training needs that can inform curriculum development and targeted interventions. The inclusion of pharmacy students from various colleges across Jordan enhances the representativeness of the findings, which could potentially improve the generalizability of the results.

However, the electronic distribution of the questionnaire made it impossible to calculate an accurate response rate. The predominance of female participants (82%) reflects the actual gender distribution in Jordanian pharmacy schools, where female enrollment typically exceeds that of males, and does not indicate a recruitment bias. The study also relies on self-reported responses, which are subject to biases such as overestimation or underestimation of knowledge and perceptions. This could affect the accuracy of conclusions about students’ understanding and confidence levels. Additionally, there may be self-selection bias, as students with a stronger interest in cancer-related topics may have been more likely to participate, which could influence the generalizability of the findings. As cross-sectional study, it captures data at a single point in time, limiting the ability to establish causal relationships. Furthermore, a defined sampling method was not used; instead, convenience sampling was employed to achieve the study objectives. Despite these limitations, our study serves as a foundational effort to address the sensitive and underexplored topic of cancer knowledge among pharmacy students in Jordan.

## Conclusion

This study found that while pharmacy students demonstrated good general knowledge of cancer and screening recommendations, their understanding of cancer risk factors, warning signs and the practical aspects of oncology care was limited. These gaps highlight the need to strengthen pharmacy curricula through enhanced oncology education and practical training opportunities. Better preparation in these areas will enable future pharmacists to play a more effective role in multidisciplinary cancer care, particularly in Jordan, where cancer incidence is rising and pharmacists are increasingly involved in prevention, treatment, and patient education.

## Supporting information

S1 File Assessment of pharmacy students’ knowledge. (PDF)
