# Supplementary material for: Assessing cancer knowledge among pharmacy students in Jordan: Bridging the gap between theory and practice
Source: PLoS One. 2025 Jul 28;20(7):e0327187. doi: 10.1371/journal.pone.0327187 (PMC12303267; doi:10.1371/journal.pone.0327187)
Supplement: S1 File — (PDF) [file pone.0327187.s001.pdf]

# Assessment of pharmacy students' knowledge about cancer and cancer recognition.

* Required

Agreement to participate

أنتم مدعون لتعبئة استبيان **يهدف إلى تقييم معرفتك حول السرطان، توصيات المسح للسرطان و شروط التحويل الى الاطباء الاخصائيين** والذي يقوم به الباحثون في كلية الصيدلة في جامعة اليرموك. من أجل المساعدة في تجويد التدريس والممارسة الصيدلانية من المتوقع أن تستغرق تعبئة وإكمال الاستبيان عشر (10) دقائق. سيتم التعامل مع جميع البيانات والمعلومات المقدمة بسرية تامة. سيتم جمع المعلومات بلا اسم ولن يتم استخدام البيانات والمعلومات المقدمة في الكشف عن هويات المشاركين. إن جميع البيانات والمعلومات المقدمة سيتم حفظها بشكل امن بالتماشي مع قواعد وتعليمات جامعة اليرموك.

You are invited to complete a survey that aims to assess your knowledge about cancer and cancer recognition that is carried out by researchers from the Faculty of Pharmacy at Yarmouk University in order to help in improving the teaching and practice. The survey completion should take a few minutes. No defined danger is caused by completion of the survey. All the survey data is confidential, participants names are not collected, which guarantees the participants' privacy. No one can know if you participated or not. Also, note that your participation in this study is voluntary, so feel withdraw from the study with no justification.

1. Do you agree to complete the suvey
هل ترغب بتعبئة الاستبيان؟ *

○ Yes نعم

○ No لا

2. Are you a Pharmacy or PharmD student? *

○ Yes

○ No

Section 1

3. What is your age? *

4. **What gender do you identify with?** *

- Male
- Female

5. **What year of your studies are you in?** *

- 1 st year
- 2nd year
- 3rd year
- 4th year
- 5th year or later

6. **university are you studying in** *

- local privte
- local public
- oversees

7. **pharmacy degree** *

- pharmD
- BSc

8. **Recieved adequate oncology education during undergraduate degree** *

- Yes
- No

9. **Received adequate chemotherapy education during undergraduate degree** *

- Yes
- No

10. **Personal history of cancer** *

- Yes
- No

11. **Family history of cancer**: *

- Yes
- No
- I do not know

12. **On a scale of 1 - 5 (1 = Lowest, 5 = Highest), In your opinion, how important for the pharmacist to know about oral chemotherapy drugs?** *

- ◯ 1 Not important
- ◯ 2
- ◯ 3
- ◯ 4
- ◯ 5 very important

13. **On a scale of 1 - 5 (1 = Lowest, 5 = Highest), how interested would you be in completing a continuing education program on oral chemotherapy?** *

- ◯ 1 Not Interested
- ◯ 2
- ◯ 3
- ◯ 4
- ◯ 5 Very Interested

14. **Have you had any teaching about oncology medication use during your undergraduate degree?** *

	Yes	No	Unsure
Pathophysiology of cancer	◯	◯	◯
Pharmacology of anticancer medicines	◯	◯	◯
Management of patient with cancer	◯	◯	◯
Safe practices to handle anticancer medicines	◯	◯	◯

15. **On which topics would you like to receive more information? (*Check all that apply.*)** *

- ☐ Pharmacology of anticancer medicines
- ☐ How to use anticacer
- ☐ Types of cancer
- ☐ Prescription of anticancer medications
- ☐ None
- ☐ Other

Section 2

16. Cancer is when abnormal cells divide in an uncontrolled way *

- ◯ True
- ◯ False
- ◯ I don't know

17. Cancer is just one disease *

- ◯ True
- ◯ False
- ◯ I don't know

18. Cancer cells can also spread to other parts of the body *

- ◯ True
- ◯ False
- ◯ I don't know

19. There are few types of cancer *

- ◯ True
- ◯ False
- ◯ I don't know

20. Cancers are usually named for the organs or tissues where the cancers form *

- True
- False
- I don't know

21. Anybody is susceptible to cancer *

- True
- False
- I don't know

22. Cancer is not life-threatening disease *

- True
- False
- I don't know

23. Cancer is an infectious disease *

- True
- False
- I don't know

24. The most common treatments for cancer are surgery, chemotherapy and radiation *

- True
- False
- I don't know

25. Early detection of cancer is difficult because there are no specific symptoms *

- True
- False
- I don't know

26. **Which of the following could be specific warning signs of cancer?** *

- ☐ A new or unusual growth, lump or swelling anywhere on the body
- ☐ Sore throat that does not heal
- ☐ Changes in the shape or size of a mole or wart
- ☐ Blood in urine or stool
- ☐ Unusual bleeding or discharge from the nipple or vagina
- ☐ Change in bowel or bladder habits
- ☐ Difficulty in swallowing
- ☐ Indigestion
- ☐ Unexplained loss in weight
- ☐ Unexplained fever, tiredness or pains
- ☐ Persistent or recurrent infection
- ☐ Joint inflammation

27. **Which of the following is considered cancer causes or risk factors?** *

- [ ] Smoking
- [ ] Radiation exposure
- [ ] Overweight and obesity
- [ ] Insufficient physical activity
- [ ] Excessive alcohol intake
- [ ] Unbalanced diet
- [ ] Vaccine
- [ ] Drink a lot of coffee
- [ ] Deodorant or antiperspirant use
- [ ] Migraines
- [ ] Infections
- [ ] Hormones
- [ ] Chronic inflammation
- [ ] Lack of fruits and vegetable intake

Section 3

28. **Which of the following cancer screening recommendations are corrrect?**

	Correct	Incorrect	Not sure
It is recommended that women aged 20 to 30 years undergo clinical breast examination periodically (every 3 years)	◯	◯	◯
Women age 40 years and above should undertake mammography annually	◯	◯	◯
Women aged 30 to 65 years should undertake HPV test and Pap test every 5 years	◯	◯	◯
Men and Women age 50 years and above should undertake faecal occult blood test annually	◯	◯	◯
Men aged 50 years at high risk should undertake Digital rectal examination annually	◯	◯	◯
At the time of menopause, women at average risk should be informed about risks and symptoms of endometrial cancer	◯	◯	◯
Women are strongly [illegible]			

encouraged to report any unexpected bleeding or spotting to their physicians ○ ○ ○
